# Proceedings of De Witt County Medical Society

**Published:** 1865-07

**Authors:** C. Goodbrake

**Affiliations:** Secretary


### PROCEEDINGS OF THE DE WITT COUNTY MEDICAL SOCIETY.

Reported by C. GOODBRAKE, M. D., Secretary.

The Society met in annual session at the office of Dr Madden, in Clinton, on the 3d day of April, the President, Dr. Hunt, in the Chair.

The Secretary being absent, Dr. Goodbrake was appointed Secretary *pro tem.*

The minutes of the last meeting were read and approved. The election of officers being in order, the following gentlemen were elected for the ensuing year: *President*, Dr. J. H. Tyler; *Vice President*, Dr. Z. H. Madden; *Treasurer*, Dr. J. B. Hunt; *Secretary*, Dr. C. Goodbrake. *Censors*, Dr. J. J. Lake, Dr. W. W. Adams, Dr. Thos. W. Davis.

*Delegates to the Illinois State Medical Society*—Dr. Benj. S. Lewis, Dr. David W. Edmistan, Dr. Joseph H. Tyler.

*Delegates to the American Medical Association*—Dr. W. W. Adams and Dr. Christopher Goodbrake.

The President elect, Dr. Tyler, on taking the Chair, made a few very happy remarks upon the importance of keeping up County Medical organizations; and his arguments proved very conclusively that Medical Associations were productive of great benefit, both to the profession and the public.

On motion, Cerebro-Spinal Meningitis was chosen as the subject for discussion at the next meeting.

Drs. Madden and Adams were appointed to write essays to be read at the next meeting.

On motion, the Secretary was ordered to furnish copies of these proceedings to the Chicago MEDICAL JOURNAL and the Chicago *Medical Examiner* for publication.

On motion, the Society adjourned to meet in quarterly session at Clinton, on the first Monday of July next.

This was a successful effort to revive the Society, which, since the war, had only met at irregular intervals. But we now look forward again to full and beneficial meetings, such as we were wont to have in days gone by.

Our Society, we believe, can boast of having furnished as large a quota for the service of our country as any other association having no more members. Dr. Evan Richards served two years and six months, and was killed at the battle of Raymond, Miss. He had attained to the rank of Lieutenant Colonel, and was at the time of his death in command of the 20th Ill. Inf. He was a brave soldier, and a "thorough read" and careful practitioner of medicine. In his death this Society lost one of its best members. Dr. B. S. Lewis was a Captain, and commanded a company in the 107th Ill. Inf., until he was compelled to resign on account of physical disability. Dr. Goodbrake served three years and four months as Surgeon of the 20th Ill. Inf., and Surgeons Wright, Ross, R. T. Richards, and Shurtleff are still in the service; but from present indications we have strong hopes that they will soon be permitted to return to their peaceful homes, and rest from their arduous duties in the field. Would it not be grand if they could all meet with us at our meeting in July next?

